# Multi-Parameter Characterization of Liquid-to-Ice Phase Transition Using Bulk Acoustic Waves

**DOI:** 10.3390/s24124010

**Published:** 2024-06-20

**Authors:** Andrey Smirnov, Vladimir Anisimkin, Natalia Voronova, Vadim Kashin, Iren Kuznetsova

**Affiliations:** 1 Kotelnikov Institute of Radio Engineering and Electronics of RAS, Moscow 125009, Russia; andre-smirnov-v@yandex.ru (A.S.); anis@cplire.ru (V.A.); vadim_kashin@mail.ru (V.K.); 2 Molecular Electronic Research Institute Stock Company, Moscow 124460, Russia; vonavl@mail.ru

**Keywords:** bulk acoustic waves, time delay, attenuation, liquid, ice, phase transition

## Abstract

The detection of the liquid-to-ice transition is an important challenge for many applications. In this paper, a method for multi-parameter characterization of the liquid-to-ice phase transition is proposed and tested. The method is based on the fundamental properties of bulk acoustic waves (BAWs). BAWs with shear vertical (SV) or shear horizontal (SH) polarization cannot propagate in liquids, only in solids such as ice. BAWs with longitudinal (L) polarization, however, can propagate in both liquids and solids, but with different velocities and attenuations. Velocities and attenuations for L-BAWs and SV-BAWs are measured in ice using parameters such as time delay and wave amplitude at a frequency range of 1–37 MHz. Based on these measurements, relevant parameters for Rayleigh surface acoustic waves and Poisson’s modulus for ice are determined. The homogeneity of the ice sample is also detected along its length. A dual sensor has been developed and tested to analyze two-phase transitions in two liquids simultaneously. Distilled water and a 0.9% solution of NaCl in water were used as examples.

## 1. Introduction

It is well known that a decrease in temperature below a certain critical value causes a first-order phase transition, which results in the transformation of a liquid into a solid (ice) [1]. The temperature at which this transition occurs depends on the type of liquid, its chemical composition, and the ambient pressure [2,3,4,5]. For most substances, the freezing process reduces the volume of the liquid, but for water, the reverse is true. This unusual behavior is due to the atomic structure of the water molecule, which consists of two hydrogen atoms and one oxygen atom. In the liquid state, water molecules are free to move around, connecting and disconnecting their hydrogen bonds. Under certain conditions, these molecules can come very close together, increasing the density of the liquid compared to that of ice. As the temperature decreases further, the motion of the water molecules slows down, and they arrange themselves in a regular lattice structure with six sides. The solid state of a substance is less dense than the liquid state because water molecules cannot stay too close to each other due to the stronger inter-molecular interactions. This is responsible for the well-known phenomenon of water expanding and destroying vessels at low temperatures [6].

The control of the water-to-ice transition is crucial for preventing damage to aircraft, ships, and roads [7]. Another important aspect of this issue is the need to monitor the state of multiphase liquid aggregation. This is significant, especially considering the potential for more intensive development of the Northern Sea Route due to climate change. Under these circumstances, strong daily temperature fluctuations can cause multiple changes in the aggregation state of different process fluids over a short period of time. Therefore, the development of new techniques and sensors for detecting the liquid-to-solid phase transition is highly relevant and greatly needed.

At present, icing control is achieved using various experimental techniques. For example, high-voltage wire control is provided using the electro-optic method [8]. Aircraft wings and air-wind power generator control are also achieved using fiber-optic [9,10], radio-frequency [11], and atomic-force technologies [12]. Ultrasonic control of the icing process has also been employed in some applications. The possibility of using ultrasound to detect the water-ice phase transition has been proposed in [13]. The work used Love surface acoustic waves (SAWs) in the “ST-quartz—*SiO*_2_ layer” structure with an operating frequency (*f*) of 234 MHz. The phase transition between water and ice on the surface of the waveguide caused a phase shift in the acoustic signal. Similar sensors based on Love SAWs in the “31YX-quartz—*SiO*_2_ layer” structure with an operating frequency of 86 MHz and in the *SiO*_2_/36YX-*LiTaO*_3_ structure with *f* = 160 MHz have been implemented [14,15]. The recent paper [16] focuses on the development of a dual-mode SAW delay line device based on a 64° rotated Y-cut *LiNbO*_3_ substrate, which allows for simultaneous ice detection and temperature measurement. The possibility of using an AT-quartz acoustic resonator with *f* = 10 MHz to study ice formation on its surface in liquid nitrogen at −193 °C is also demonstrated [17]. The possibility of using acoustic waves with shear-horizontal polarization and higher-order Lamb waves for detecting the water-to-ice phase transition on the surface of metal plates has been demonstrated both theoretically and experimentally [18,19]. These waves were generated using electromagnetic acoustic transducers (EMATs) [18,19]. The feasibility of detecting such phase transitions with waves in piezoelectric materials has been shown in [20,21,22]. A benefit of using these acoustic waves for ice sensing is that it allows for the placement of liquid on the plate surface, which is free of electrodes [23].

Furthermore, the icing on the surface of the layered plate is detected using a Lamb wave tomography technique and an array of piezoelectric elements positioned on the plate’s surface [24]. When the properties of any part of the surface change due to icing, the output signals from the relevant piezoelements change, and the sensor not only detects the icing process, but also the coordinates of the icing area.

The freezing of a thin water film with a thickness of 40 microns was analyzed using reflected ultrasonic echo pulses and a relevant numerical model [25]. This approach allowed us to measure the thickness of the glaze ice.

In general, all the papers mentioned above demonstrate the usefulness of ultrasonic techniques for studying liquid-to-ice phase transitions. The works are mainly focused on the study of ice formation on flat surfaces and analyzes the surface covered by one type of liquid (water) or ice. However, it is an important practical challenge to develop methods for controlling ice formation in volume.

This paper proposes a number of experimental techniques for studying liquid-to-ice phase transitions, based on the fundamental properties of bulk acoustic waves [26,27,28]. The bulk acoustic waves with shear vertical or shear horizontal polarization cannot propagate in liquids, only in solids, such as ice. However, longitudinal polarization waves can propagate in both liquids and solids, with different velocities and attenuations depending on the aggregate state of matter. The techniques developed make it possible to track the phase transition process in real-time for both one liquid sample and two samples simultaneously. They also allow us to assess the uniformity of ice thickness and measure its acoustic properties.

Another challenge is measuring the velocity and attenuation of Rayleigh surface acoustic waves (SAWs), which occur, for example, due to an earthquake. As is known [29], the characteristics of these waves depend strongly on ice parameters, and they can be used to monitor changes in the firn properties and the thermal composition of Greenland and Antarctic ice sheets under a changing climate. Currently, information about these waves is obtained from ambient noise recordings using degree-of-polarization (DOP) analysis [30]. The results in this paper allow us to determine the velocity and attenuation of Rayleigh SAWs in ice, as well as the Poisson’s modulus for ice, using relevant bulk acoustic wave (BAW) properties.

## 2. Materials and Methods

### 2.1. Design of a BAW Sensor for Registration of Liquid-to-Ice Phase Transition

**Figure 1:**
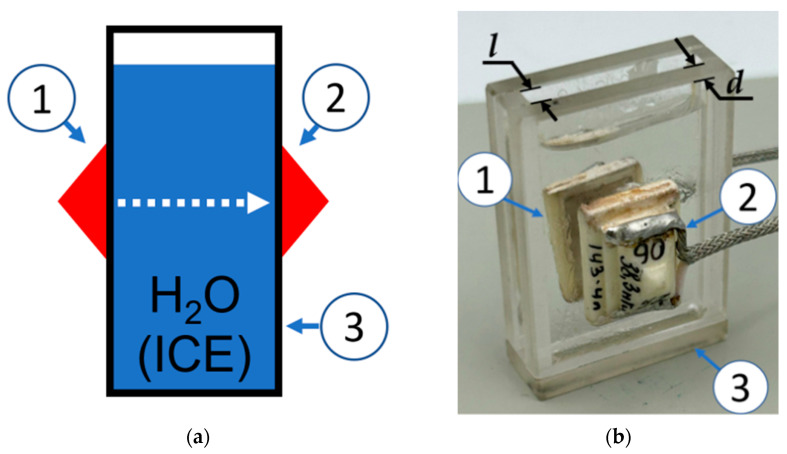
(**a**) Schematic view and (**b**) photo of the BAW sensor for liquid–ice phase transition with input transducer (1), output transducer (2), and cell (3).

Figure 1 shows a schematic view (a) and a photo (b) of the sensor for registering of the liquid–ice phase transition using BAW. It consists of an input transducer (1), an output transducer (2) and a cell (3) for testing liquid or ice. Z-cut *LiNbO_3_* (3 × 4 mm^2^ in area) operating at *f* = 3–30.9 MHz were used as the LBAW transducers (JSC “Research Institute “ELPA”, Moscow, Russia). YX quartz (8 × 13 mm^2^ in area) operating at *f* = 10–37 MHz served as the SVBAW transducers (JSC “Research Institute “ELPA”, Moscow, Russia). The transducers were glued to the cell using epoxy resin, which provided good acoustic contact over a temperature range of −25 °C to +60 °C. The cells were made from fused quartz with a wall thickness of *d* = 2.5–2.7 mm and a wall-to-wall distance of *l* = 3–17.5 mm. The material constants of fused quartz were taken from [31]. It should be noted that as the distance *l* increases, the perturbation of the test sample by the cell walls decreases. However, at the same time, acoustic attenuation in the test medium also increases with increasing distance *l*. Therefore, each measurement requires a careful balance between the liquid perturbation and the wave attenuation.

The BAW sensor (Figure 1) was mounted in thermal camera (UC-20CE, Taiwan) proving temperature (*T*) variations from +20 °C to −20 °C with 1 °C step. The input and output transducers were connected with the Network Analyzer KeySight E5061B (Keysight, Santa Rosa, CA, USA), operating in an amplitude–time format *S_12_*(*τ*). *S_12_* and *τ* are transfer function (insertion loss) and time, respectively. The use of a network analyzer in our ultrasonic measurements is justified by its ability to average the measured data and reduce noise. This is especially important for analyzing phase transitions, which are accompanied by increased BAW attenuation, and for high-temperature measurements where epoxy resin, used as a glue, is softened. In order to prevent damage to the liquid cell caused by ice growth, two wooden sticks with a diameter of 0.5 mm and a length of 60 mm were inserted into the test liquid before the water began to freeze. The sticks were placed outside of the acoustic wave propagation path. The change in the path length *l* between the transducers due to thermal expansion of the cell was negligible (1/*d*(∂*d*/∂*T*) + 1/*l*(∂*l*/∂*T*)~100 ppm/°C).

### 2.2. Design of a BAW Sensor for Ice Homogeneity Control

**Figure 2:**
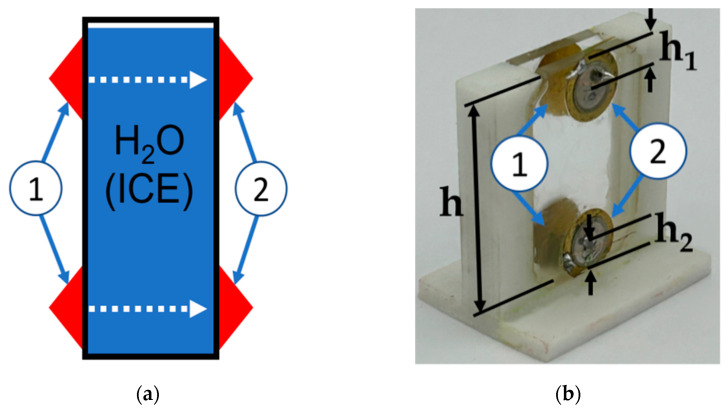
(**a**) Schematic view and (**b**) photo of the LBAW sensor for detecting ice homogeneity: input LBAW transducers (1), output LBAW transducers (2), and cell for ice sample at *h* = 30 mm, *h_1_* = 4 mm, *h_2_* = 4 mm, *f_L_* = 13 MHz, *l* = 17.8 mm, and *d* = 3.1 mm.

The design of the BAW sensor for ice homogeneity control is shown in Figure 2. It consists of two input transducers (1) and two output transducers (2), as well as a cell for the ice sample. The homogeneity of the ice sample along its length was experimentally examined by comparing the velocity and attenuation of the LBAW signals measured at the top and bottom ends of the sample (Figure 2). Thickness-polarized PZT-17 transducers with a diameter of 10.5 mm and *f* = 13 MHz were used as the LBAW transducers (JSC “Research Institute “ELPA”, Moscow, Russia). The cell was made from fused quartz with a wall thickness of *d* = 3.1 mm and wall-to-wall distance of *l* = 17.8 mm.

### 2.3. Design of a Dual BAW Sensor for Simultaneous Detection of Two Liquid-to-Ice Transformations

**Figure 3:**
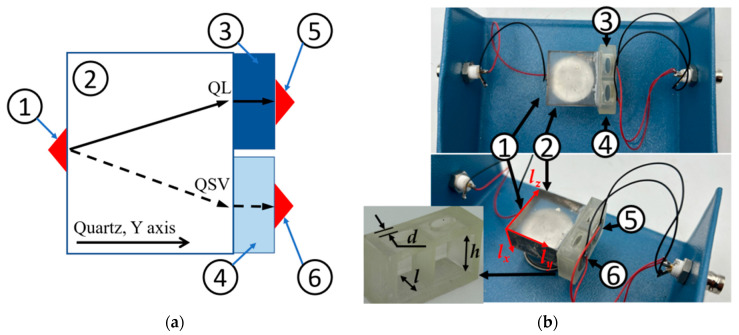
(**a**) Schematic view and (**b**) photo of the dual BAW sensor for simultaneous detection of two liquid-to-ice transitions: input LBAW transducer (1), bar quartz substrate (*l_z_* = *l_y_* = 32 mm, *l_x_* = 16 mm) (2), two cameras of Teflon cells for liquid 1 and liquid 2 or for ice (3, 4), two output LBAW transducers (5, 6) at *d* = 1 mm, *l* = 7 mm, *h* = 15 mm.

The schematic view (a) and photo (b) of the dual BAW sensor are shown in Figure 3. The sensor consists of an input transducer (1), a bare quartz crystal (2), a Teflon cell with two isolated chambers, (3) and (4), without a wall between them and the quartz crystal, and two output transducers, (5) and (6), glued to opposite wall of the cell. The dimensions of the quartz crystal are 32 mm in the Z and Y directions (*l_z_* = *l_y_* = 32 mm), and 16 mm in the X direction (*l_x_* = 16 mm). The height *h* of the chambers (3) and (4) is 16 mm. The transducers are made of polarized piezo-ceramics PZT-17, which vibrates at *f* = 13 MHz in the longitudinal direction (JSC “Research Institute “ELPA”, Moscow, Russia). The thickness of the chamber walls *d* is 1 mm, and the distance *l* between end of the quartz crystal and the chamber wall (the length of the test medium along the wave propagation direction) is 7 mm. The chambers were sealed using epoxy resin. One chamber was filled with distilled water, while the other one was filled with a 0.9% solution of *NaCl* for testing purposes.

The operation of the sensor is as follows: The transducer (1) generates in the Y-quartz crystal (2) two BAWs. One of them has the greater longitudinal component of displacement (*u_y_*) and is named quasi-longitudinal (QL) BAW (*V_QL_* = 6010 m/s, {*u_x_* = 0, *u_y_* = 0.91, *u_z_* = 0.42}). The other one has a greater shear component of displacement (*u_z_*) and is named quasi-shear (QSV) BAW (*V_QSV_* = 4350 m/s, {*u_x_*= 0, *u_y_* = 0.42, *u_z_* = −0.91}) [32]. The power flows of these waves are steered away from the wave normal in opposite directions (*Ψ_QL_*= +23°, *Ψ_QSV_*= −24°) (Figure 3a). This results in the geometrical separation of these two acoustic beams in quartz crystal (Figure 3a). After passing through the quartz crystal, each beam travels through its own camera (3) or (4) and is received by its own output transducer (5) or (6). The 3rd quasi-shear BAW existing in quartz crystal is not generated in the Y-direction because its polarization {*u_x_* = 1, *u_y_* = 0, *u_z_* = 0} is orthogonal to the polarization of the input transducer (1) {*u_x_* = 0, *u_y_* = 1, *u_z_* = 0}.

When the QL and QSV beams reach chambers (3) and (4), the waves transform into their pure longitudinal and pure shear counterparts, as the liquids or ice inside the cameras are isotropic. The pure longitudinal waves that propagate through medium 1 in chamber 3 and medium 2 in chamber 4 are then recorded by output transducers 5 and 6, respectively, which have longitudinal polarization. At the same time, the two pure shear waves that propagate in the same ice medium and chamber are not detected by transducers 5 and 6, as these waves and the transducers have mutually orthogonal polarizations {*u_x_* = 1, *u_y_* = 0, *u_z_* = 0} and {*u_x_* = 0, *u_y_* = 1, *u_z_* = 0}.

The accuracy of the measurements was estimated to be 0.1 μs for delay time, 5% for wave velocity, and 0.5 dB for wave attenuation. It is defined as an average of 5 separate measurements, where the equipment and sample are disconnected completely between each measurement and then reconnected. Therefore, it automatically includes all measurement errors that arise from the network analyzer, transducers, glue, and temperature.

## 3. Results and Discussion

### 3.1. Registration of Liquid-to-Ice Phase Transition Using the Developed BAW Sensor

**Figure 4:**
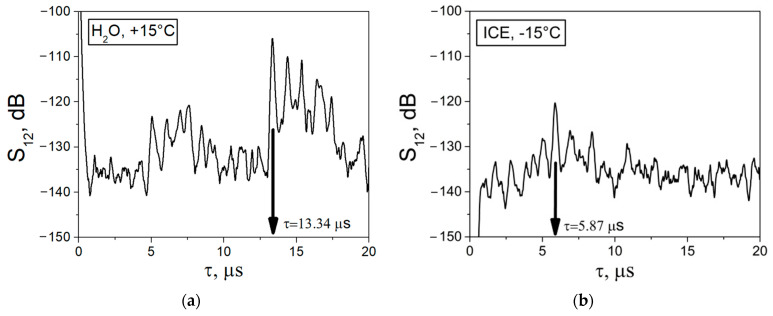
The dependences of the insertion loss *S_12_* vs. time delay *τ* measured with LBAW in distilled water (**a**) before (*T* = +15 °C) and (**b**) after (*T* = −15 °C) phase transition. *f_L_* = 30 MHz, *l* = 17.5 mm, *d* = 2.5 mm.

By using the developed BAW sensor (Figure 1) the dependence of the insertion loss *S_12_* on the time delay *τ* of the LBAW was measured for distilled water and ice before (*T* = +15 °C) and after (*T* = −15 °C) the phase transition. The results of these measurements are presented in Figure 4. The first signal from the left is electromagnetic leakage, traveling from input to output transducers at the velocity of light. The second signal from the left is an acoustic wave, propagating from the input to the output transducers at the velocity of sound. Other signals, with larger delays, are LBAWs that reflect back and forth within the cell walls. Since LBAW can propagate through both liquid and ice, the signals corresponding to these waves are recorded in both media. As the velocities of the LBAWs in ice and water are different ($V_{L}^{H_{2}O}$ < $V_{L}^{ice}$), the time delays *τ* are also different ($\tau_{L}^{H_{2}O}$ > $\tau_{L}^{ice}$). So, as can be seen in Figure 4 for water at *T* = +15 °C, the LBAW signal is registered at *τ_L_* = 13.34 μs, while for ice at *T* = −15 °C the same signal is recorded at *τ_L_*= 5.87 μs. The jump in the time delay *τ_L_* produced by the water-to-ice transformation is as large as Δ*τ_L_* = 7.5 μs.

Also, it has been found that the attenuation of LBAW in polycrystalline ice ($S_{12}^{ice}$ = 120 dB) is higher than that is in water ($S_{12}^{H_{2}O}$ = 105 dB) (Figure 4). So that, when the temperature is decreased, the LBAW signal not only shifts from one time position to another, but also changes in amplitude. For intermediate temperatures from 0 °C to −5 °C, when the test medium is a mixture of ice and water components, the LBAW signal is not visible due to excessive acoustic attenuation and propagation losses.

The LBAW velocities in water $V_{L}^{H_{2}O}$ and ice $V_{L}^{ice}$ were calculated from the distance *l* between the cell walls and the time delays measured for the cells containing water $\tau_{L}^{H_{2}O}$ and ice $\tau_{L}^{ice}$, respectively. These calculations took into account the time delays caused by the two cell walls $\tau_{L}^{SiO_{2}}=2d/V_{L}^{SiO_{2}}$:

(1) $$V_{L}^{H_{2}O}=l/\left(\tau_{L}^{H_{2}O}-\tau_{L}^{SiO_{2}}\right),\;V_{L}^{ice}=l/\left(\tau_{L}^{ice}-\tau_{L}^{SiO_{2}}\right).$$

The LBAW attenuation in water $\alpha_{L}^{H_{2}O}$ and ice $\alpha_{L}^{ice}$ were calculated as follows:

(2) $$\alpha_{L}^{H_{2}O}=\left(S_{12_{L}}^{H_{2}O}-S_{12_{L}}^{SiO_{2}}\right)/l;\;\alpha_{L}^{ice}=\left(S_{12_{L}}^{ice}-S_{12_{L}}^{SiO_{2}}\right)/l.$$

where $S_{12_{L}}^{H_{2}O}$ and $S_{12_{L}}^{ice}$ are the LBAW insertion losses measured between the input and the output transducers for water and ice, respectively. $S_{12_{L}}^{SiO_{2}}$ is the combined loss, which includes transduction losses in the two transducers, losses in the two glue layers, and losses in the two cell walls. Theses combined losses were measured independently at *T* = +15 °C ($S_{12_{L}}^{SiO_{2}}$ = 72 dB) and *T* = −15 °C ($S_{12_{L}}^{SiO_{2}}$= 54 dB) using the cell without water, the same transducers and the same glue layers. Similarly, the jump in the insertion loss caused by the water-to-ice transition (Figure 4), like the time delay *τ_L_* is mainly due to a change in propagation losses ($\alpha_{L}^{ice}$ − $\alpha_{L}^{H_{2}O}$) × *l* = (3.8–1.9) dB/mm × *l* = 33.5 dB (Table 1) rather than to changes in the transducers, glue layers, or cell walls $S_{12_{L}}^{SiO_{2}}$ (*T* = +15 °C) − $S_{12_{L}}^{SiO_{2}}$ (*T* = −15 °C) = 72–54 = 18 dB.

A key point of the whole strategy described above is that the temperature dependences of the time delay *τ_L_* and combined losses $S_{12_{L}}^{SiO_{2}}$ for empty cell are both flat linear functions with no jumps. Therefore, the appearance of jumps in *τ_L_* and *α_L_* can be attributed solely to the water-to-ice transition that occurs in the space between the two fused quartz walls of the cell.

**Figure 5:**
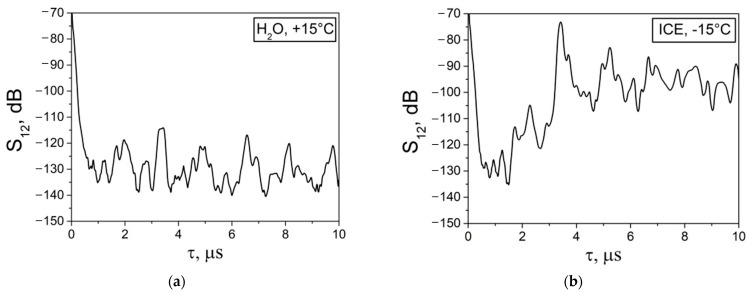
The dependences of the insertion loss *S_12_* vs. time delay *τ* measured for SVBAW in distilled water (**a**) before (T = +15 °C) and (**b**) after (T = −15 °C) phase transition at *f_SV_* = 25 MHz, *l* = 3.3 mm, *d* = 2.7 mm.

Similar results for SVBAW are presented in Figure 5 and Table 2. The procedures of the measurements with LBAW and SVBAW and parameters calculation were similar.

As mentioned above, SVBAW cannot propagate in a liquid medium and the acoustic signal for water is absent (Figure 5a). The signal only appears for solid ice (Figure 5b). The combined loss $S_{12_{SV}}^{SiO_{2}}$ value measured at *T* = −15 °C for two transducers, two glue layers (salol), and two cell walls was 53 dB.

The experimental results measured with LBAW and SVBAW using the developed method are in agreement with those reported in previous papers [33,34,35].

The measured data shows that after the water-to-ice phase transition the LBAW velocity $V_{L}^{ice}$ and attenuation $\alpha_{L}^{ice}$ increase compared to relevant values $V_{L}^{H_{2}O}$, and $\alpha_{L}^{H_{2}O}$ before the transition. We believe that this increase in velocity can be explained by the stronger interatomic forces and larger elastic moduli of ice compared to those of water. The increase in attenuation can be attributed to wave scattering by the polycrystalline structure of ice, as the dimensions of the crystallites (~1 mm) are larger than the acoustic wavelength (50–100 μm) at 30 MHz.

Further steps for ice characterization were taken using the as-measured data $V_{L}^{ice}$, $V_{SV}^{ice}$, $\alpha_{L}^{ice}$, and $\alpha_{SV}^{ice}$, assuming that ice is an isotropic medium because of its chaotic orientation of individual crystallites. The Poisson’ module *σ^ice^* for ice was determined from $V_{L}^{ice}/V_{SV}^{ice}$ ratio [36]. The Rayleigh SAW velocity $V_{R}^{ice}$ in ice was found as $V_{R}^{ice}=\xi \left(\sigma^{ice}\right)\times V_{SV}^{ice}$, where *ξ* is the function of the Poisson’ module *σ^ice^* [36]. The SAW attenuation in ice $\alpha_{R}^{ice}$ was estimated by using the following equation:

(3) $$\alpha_{R}^{ice}=A\times \alpha_{L}^{ice}+\left(1-A\right)\times \alpha_{SV}^{ice},$$

where *A* is another function of the *σ^ice^* [37]. The obtained results are presented in Table 3.

The data obtained show that the LBAW and Rayleigh wave attenuation ($\alpha_{L}^{ice},\alpha_{R}^{ice})$ are significantly lower than those for SVBAW ($\alpha_{SV}^{ice}$) at approximately the same frequency (Table 1, Table 2 and Table 3). Therefore, the inter-connection between ice crystallites in the longitudinal direction should be, on average, stronger than in the vertical direction. The velocities of the BAWs measured in ice are in the correct ratio to each other: $V_{L}^{ice}$ > $V_{SV}^{ice}$ > $V_{R}^{ice}$.

### 3.2. Ice Homogeneity Control Using the Developed LBAW Sensor

**Figure 6:**
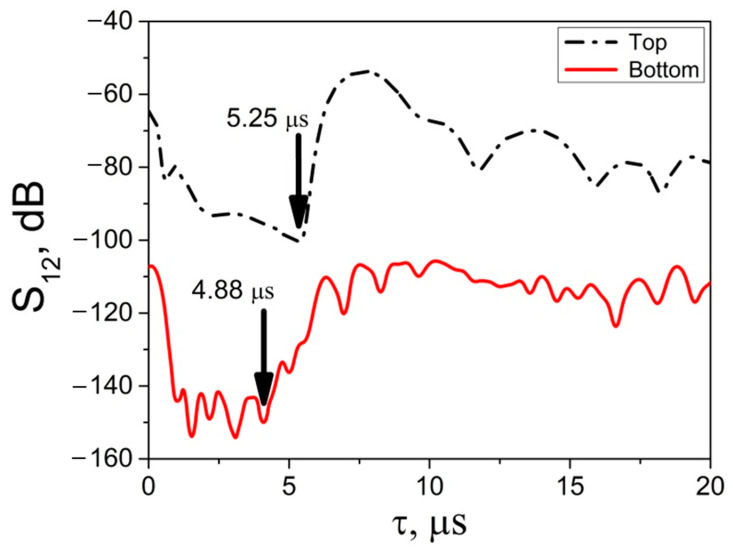
Insertion loss *S_12_* vs. time delay *τ* measured at top (black line) and bottom (red line) ends of ice sample at *T* = −20 °C, *h* = 30 mm, *h_1_* = 4 mm, *h_2_* = 4 mm, *f_L_* = 13 MHz, *l* = 17.8 mm, *d* = 3.1 mm, and $S_{12_{L}}^{SiO_{2}}$ = 37.5 dB (top) and 42.5 dB (bottom).

The possibility of determining the uniformity of the ice sample by its height was tested by using the developed LBAW sensor (Figure 2). For this purpose, measurements of insertion losses and delay time in the upper and lower parts of the ice sample at *T* = −20 °C were carried out using transducers, located respectively (Figure 2). The resulting dependencies are shown in Figure 6. Table 4 presents the calculated values for the velocity $V_{L}^{ice}$ and attenuation $\alpha_{L}^{ice}$ of the LBAW. It was found that differences in the wave velocity and the attenuation can reach up to 12% and 500%, respectively, depending on where the characteristics of an ice sample are measured (Table 4). It is evident that the highest values for $V_{L}^{ice}$ and $\alpha_{L}^{ice}$ observed in the lower part of an ice sample.

We believe that this difference is due to the different conditions of the phase transition. Indeed, when the temperature decreases, the lower layer of ice forms in contact with the walls and bottom of the cell, and the upper layer of the formed ice. At the same time, the upper layer of ice is in contact with the cell walls, the lower layer of the formed ice, and the air. These different boundary conditions during the phase transition lead to the formation of ice with different crystallographic structures and different physical properties [38].

Our hypothesis is supported by the different shapes of the *S_12_*(*τ*) curves measured using the same LBAW for ice grown in different cells. Depending on the geometry of the cells, ice can have one or another crystallographic structure and physical properties. Depending on the structure and properties, the ice samples demonstrate different *S_12_*(*τ*) curves, as seen from the comparison of the data in Figure 4b and Figure 6.

### 3.3. Simultaneous Detection of Two Liquid-to-Ice Transformations Using the Dual BAW Sensor Developed

**Figure 7:**
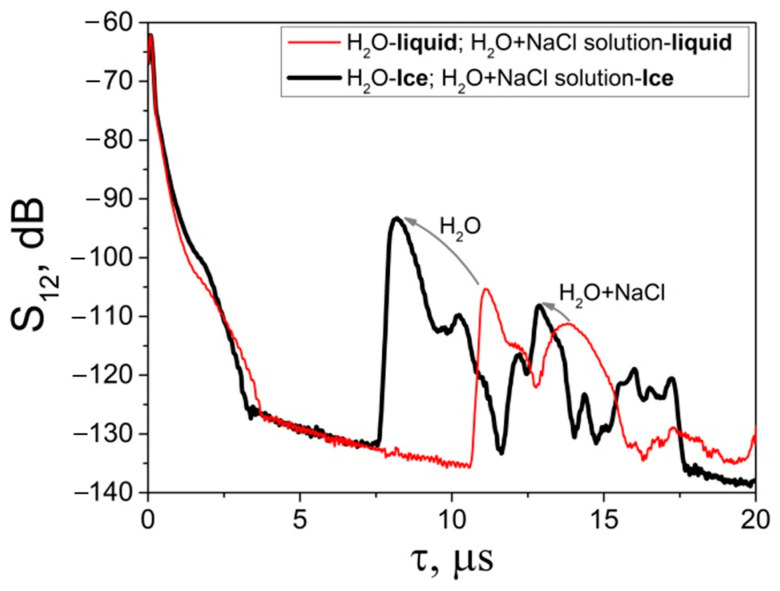
Dependence of the insertion loss *S_12_* vs. time delay *τ* measured in dual BAW sensor for two liquids and two ices simultaneously. The liquids at *T* = +20 °C (red line) and the ice samples at *T* = −20 °C (black line). The arrows indicate shifts of the QL and QSV signals due to liquid-to-ice phase transitions at *d* = 1 mm, *l* = 7 mm, *h* = 15 mm, and *f_L_* = 13 MHz.

The dependence of insertion loss *S_12_* on time delay *τ* measured for the dual BAW sensor developed (Figure 3) is shown in Figure 7. The delay time for each signal is the sum of the partial delays acquired by the wave in the quartz crystal (32 mm), the test medium (7 mm), and the cell walls (1 mm). The total delay time calculated from the sum of partial delays agrees with the experimental data within experimental error (Table 5). Similarly, the insertion loss in each channel can be expressed as the sum of losses in transducers, glue layers (epoxy), and the test medium. Specifically, the decrease in insertion loss S_12_ for ice samples at a negative temperature (Figure 7) can be attributed to improved epoxy contacts between the transducers and the quartz crystal, as well as between the transducers and Teflon cells.

## 4. Conclusions

A method based on BAW for sensing liquids, ice, and liquid-to-ice transitions has been modified and used. The method is based on measuring the time delay and attenuation of bulk acoustic waves that propagate through a test sample at different temperatures. When the aggregate state of the sample changes, both acoustic parameters change, causing abrupt shifts.

The BAW velocities and attenuations in water and ice are measured. The water-to-ice phase transition is detected, and the inhomogeneity of the ice sample along its length is recorded. Structural differences between various ice samples are sensed, and the two-phase transitions for two different liquids are measured simultaneously. This method, based on BAW, can be considered complementary to other acoustic methods, such as those based on Lamb waves.

The disadvantage of the BAW method is its reliance on the operation of transducers and on the quality of contacts between the transducer and the substrate, which can change with temperature. This means that each sensor must be calibrated individually to account for these variations. The precision of measurements is estimated to be ±0.1 μs for time delay, ±5% for velocity, and ±0.5 dB/mm for attenuation.

Such methods and sensors could be useful for the scientific study of water, ice, and the water-to-ice phase transition in different Arctic regions under varying weather conditions.

## Figures and Tables

**Table 1:** Phase velocities *V_L_*, delay times *τ_L_*, and attenuations *α_L_* measured with LBAW in H_2_O (*T* = +15 °C) and in ice (*T* = −15 °C) at *f_L_* = 30 MHz, *l* = 17.5 mm, and *d* = 2.5 mm.

	$\tau_{L}^{H_{2}O}$, μs	$V_{L}^{H_{2}O}$, km/s	$\alpha_{L}^{H_{2}O}$, dB/mm	$\tau_{L}^{ice}$, μs	$V_{L}^{ice}$, km/s	$\alpha_{L}^{ice}$, dB/mm	$\tau_{L}^{SiO_{2}}$, μs	$V_{L}^{SiO_{2}}$, km/s
Present paper	13.34	1.4 ± 0.1	1.9 ± 0.5	5.87	3.5 ± 0.2	3.8 ± 0.5	0.84	5.96 ± 0.1
Other papers	-	1.466 ± 0.003 [33]	-	-	3.6 ± 0.1 [34]	-	-	5.97 [35]

**Table 2:** Phase velocities *V_SV_*, delay times *τ_SV_*, and attenuations *α_SV_* measured for SVBAW in ice at *T* = −15 °C, *f_SV_* = 25 MHz, *l* = 3.3 mm, *d* = 2.7 mm.

	$\tau_{SV}^{ice}$, μs	$V_{SV}^{ice}$, km/s	$\alpha_{SV}^{ice}$, dB/mm	$\tau_{SV}^{SiO_{2}}$, μs	$V_{SV}^{SiO_{2}}$, km/s
Present paper	3.4	1.7 ± 0.2	6.1 ± 0.5	1.44	3.76 ± 0.1
Other papers	-	1.929 ± 0.02 [34]	-	-	3.76 [35]

**Table 3:** Ice properties measured in present paper.

$V_{L}^{ice}$, km/s	$V_{SV}^{ice}$, km/s	$V_{R}^{ice}$, km/s	$\alpha_{L}^{ice}$, dB/mm	$\alpha_{SV}^{ice}$, dB/mm	$\alpha_{R}^{ice}$, dB/mm	*σ^ice^*
3.5 ± 0.2	1.7 ± 0.2	1.6 ± 0.2	3.8 ± 0.5	6.1 ± 0.2	4 ± 0.5	0.35 ± 0.04

**Table 4:** Results of the measurements at top and bottom ends of ice sample according to Figure 6 at *h* = 30 mm, *h_1_* = 4 mm, *h_2_* = 4 mm, *f_L_* = 13 MHz, *l* = 17.8 mm, *d* = 3.1 mm, *T* = −20 °C.

Transducers Position	$V_{L}^{ice}$, km/s	$\alpha_{L}^{ice}$, dB/mm
Top	4.2 ± 0.2	0.7 ± 0.2
Bottom	4.7 ± 0.2	3.8 ± 0.5

**Table 5:** Results of the measurements according to Figure 7.

Liquid Phase			Solid Phase		
	*H_2_O*	*H_2_O* + 0.9% *NaCl*		Pure Ice	Salty Ice
*τ_lq_*, μs	10.2 ± 0.5	11.8 ± 0.5	*τ_ice_*, μs	7.5 ± 0.5	11.2 ± 0.5
$V_{L}^{lq}$*,* m/s	1500	1100	$V_{L}^{ice}$, m/s	3500	1250

## Data Availability

No new data were created or analyzed in this study. Data sharing is not applicable to this article.
